# BMI, waist circumference at 8 and 12 years of age and FVC and FEV_1_ at 12 years of age; the PIAMA birth cohort study

**DOI:** 10.1186/s12890-015-0032-0

**Published:** 2015-04-22

**Authors:** Marga B Bekkers, Alet H Wijga, Ulrike Gehring, Gerard H Koppelman, Johan C de Jongste, Henriette A Smit, Bert Brunekreef

**Affiliations:** Institute for Risk Assessment Sciences (IRAS), Utrecht University, Utrecht, The Netherlands; Centre for Nutrition, Prevention and Health Services, National Institute for Public Health and the Environment (RIVM), Bilthoven, The Netherlands; Department of Pulmonology and Pediatric Allergology, GRIAC Research Institute, University of Groningen, University Medical Center Groningen, Groningen, The Netherlands; Department of Pediatrics, Erasmus University Medical Center, Sophia Children’s Hospital, Rotterdam, The Netherlands; Julius Centre for Health Sciences and Primary Care, University Medical Center, Utrecht, The Netherlands

**Keywords:** Birth cohort, Lung volume, Overweight

## Abstract

**Background:**

In adults, overweight is associated with reduced lung function, in children evidence on this association is conflicting. We examined the association of body mass index (BMI) and waist circumference (WC) at age 12, and of persistently (at ages 8 and 12 years) high BMI and large WC, with forced vital capacity (FVC) and forced expiratory volume in 1 second (FEV_1_) at age 12.

**Methods:**

Height, weight, WC and FVC and FEV_1_ were measured during a medical examination in 1288 12-year-olds participating in the PIAMA birth cohort study. 1090 children also had BMI and WC measured at age 8. The associations between BMI and WC and FVC, FEV_1_, and FEV_1_/FVC ratio were studied using local and linear regression analyses, separately for girls and boys. The regression models were adjusted for age, height, and pubertal development and maternal educational level.

**Results:**

High BMI and large WC (sd-score >90th percentile) were associated with higher FVC; in girls these associations were statistically significant (4.6% (95% CI: 1.5, 7.9) and 3.6% (95% CI: 0.6, 6.8) respectively in adjusted models). Similar associations were observed for persistently high BMI or large WC: girls with a high BMI or large WC at both 8 and 12 years had statistically significantly higher FVC at age 12 years (BMI: 4.9% (95% CI 0.9, 9.1), WC: 5.0% (95% CI 0.7, 9.6)) than girls with normal BMI or WC at both ages. No statistically significant associations were observed between (persistently) high BMI or large WC and FEV_1_. The FEV_1_/FVC ratio was statistically significantly lower in children with a high BMI or large WC than in children with a normal BMI or WC. Girls and boys with a persistently high BMI or large WC status had statistically significantly lower FEV_1_/FVC ratios.

**Conclusion:**

At 12 years of age, a persistently high BMI or large WC is not yet associated with lower FVC and FEV_1_, suggesting that this association, that is commonly observed in adults, develops at a later age.

**Electronic supplementary material:**

The online version of this article (doi:10.1186/s12890-015-0032-0) contains supplementary material, which is available to authorized users.

## Background

Lung diseases contribute substantially to mortality [1]. Lung function deficits in childhood may increase the risk of lung diseases later in life. Smoking parents, genetic predisposition and air pollution have been shown to negatively influence children’s lung function [2,3]. A relatively new potential risk factor for lower lung function in children is overweight. In adults, being overweight, measured by BMI, is associated with reduced lung function [4-8]. In children, the prevalence of overweight has increased considerably but studies in children on the association between overweight and lung function show inconsistent results. In contrast to studies in adults, most studies in children seem to suggest a positive association [9-13] rather than an inverse association [14] between BMI and forced vital capacity (FVC) and forced expiratory volume in 1 second (FEV_1_). The observation of different directions of the association of BMI with lung function between adults and children suggests that the association between adiposity and lung function changes with age. However, the age at which this change of direction occurs is not known more specifically than at some time during adolescence. Previously, in 8-year-old children we did not observe lower FVC, and FEV_1_ in children with a high BMI [15]. Therefore, in the current study we investigate the association of BMI and waist circumference (WC) with FVC, FEV_1_ and FEV_1_/FVC in the same cohort of children 4 years later, i.e. at age 12 years, to examine whether the change in the association between BMI and lung function already becomes apparent around that age. Moreover, persistence of overweight might play a role in the association of BMI and WC with lung function. Possibly, a longer period of excess fat mass may negatively affect FVC and FEV_1_ and the ratio between those two. That is why we additionally aim to study the importance of persistence of high BMI and large WC in the association between adiposity and FVC, FEV_1_ and FEV_1_/FVC ratio.

## Methods

### Study design and population

The children in this study are participants of the Dutch Prevention and Incidence of Asthma and Mite Allergy (PIAMA) birth cohort study and were born in 1996–1997. This research was performed in accordance with the ethical principles for medical research involving human subjects outlined in the Declaration of Helsinki. Therefore, the study protocol was approved by the Medical Ethics Committees of the participating institutes (Rotterdam, start project MEC 132.636/1994/39 and 137.326/1994/130; Rotterdam 8 years MEC 2004–152; Groningen, start project MEC 94/08/92; Groningen 8 years M 4.019912; Utrecht, start project MEC-TNO oordeel 95/50; Utrecht 8 years 04-101/K; Utrecht 12 years 07–337/K) and by the Dutch Central Committee on Research involving Human Subjects (8 years P04.0071C; 12 years 07-337/K). All parents and the children gave written informed consent. The initial objectives of the study were to evaluate the effectiveness of allergen reduction measures for the prevention of asthma and mite allergy in children of allergic mothers, and to investigate the natural history of childhood asthma and risk factors for the development of asthma. Later, the study aims were expanded to include early markers of cardiovascular disorders. A detailed description of the study design has been published previously [16]. At baseline, the cohort consisted of 4146 pregnant women, 183 being lost to follow-up before any data of the child had been collected, so that the study started with 3963 newborns. At the age of 12 years 3541 children were still in the study, and were invited for the medical examination (n = 3202), except children that moved abroad or too far from the study regions and children with mental or behavioral limitations. 1511 children (47.1%) agreed to participate. Finally, the medical examination, including anthropometric measurements and lung function tests, was performed in 1432 children (94.8%). In 1288 children (89.9% of 1432) the lung function tests met our inclusion criteria. Out of these children, 1090 (84.6%) also participated in the medical examination of 8-year-olds and were included in the analyses of BMI and WC at ages 8 and 12 years.

### Height, weight, waist circumference and lung function

All anthropometric variables were measured while the children were wearing underwear only. Weight was measured at the nearest 0.1 kg and height (cm) was measured at one decimal. BMI was calculated as weight in kilograms divided by height squared in meters (kg/m^2^). WC (cm) was measured twice at one decimal and the mean of the two waist measurements being used in analyses. Additionally, BMI and WC sd-scores (standard deviation) for age and gender were calculated using the reference growth curves of the Dutch Fourth nationwide Growth study [17]. We then divided the children in three BMI sd-score categories and into three WC sd-score categories: 1) below the 10th percentile, 2) above the 90th percentile and 3) between the 10th and 90th percentile, which we call ‘normal’. The 10% children in our study population with the lowest sd-scores for BMI and WC were defined as having a low BMI and small WC and the 10% children in our study population with the highest sd-scores for BMI and WC were defined as having a high BMI and large WC. The percentiles were based on the distribution for boys and girls separately. Besides height, also sitting height was measured.

As outcome measures we used FVC as a measure of lung volume, FEV_1_ as a measure of airway patency and the FEV_1_/FVC ratio to indicate possible obstruction of the airways. An EasyOne spirometer (NDD Medical Technologies, USA) was used for lung function testing. The machines were calibrated every day the medical examination took place. FVC and FEV_1_ were measured in sitting position, while wearing a nose clip, according to the ATS/ERS guidelines [18]. Following the ATS/ERS criteria [18] we included measurements if at least three acceptable maneuvers had been obtained. Also test results were included which were obtained from otherwise technically acceptable flow-volume curves with the two largest FEV_1_ within 200 mL difference, but which did not completely meet the before mentioned criteria. All measurements during the medical examination were performed by trained research staff using calibrated measuring equipment.

### Covariates

Data on covariates were collected during the medical examination and in the yearly questionnaires. In the questionnaires, data on the child’s pubertal development (using the pubertal development scale [19]), tobacco smoke exposure and gas cooking and the education level of the mother were collected. The latter was measured as the highest education completed and divided into three categories; low, intermediate and high education. During the medical examination including the lung function test, the parents were interviewed on their child’s current health complaints and medication use. Blood sampling of the children was performed for measurement of specific IgE concentrations. Sensitization to allergens was defined as having a specific IgE concentration of ≥ 0.70 IU per mL against one or more of the following allergens: house dust mite, cat, grass (*Dactylis glomerata*), and birch pollen.

### Statistical analyses

In the analyses of the association of BMI and WC with FVC, FEV_1_ and FEV_1_/FVC ratio the natural logarithm of the lung function testing variables and of height and age were used to capture the complex and non-linear association between these variables [20]. The analyses were stratified by gender because lungs develop differently in boys and girls [21]. Sitting height or the ratio between sitting height and total height might be more important in association with lung function than ‘total’ height [22]. Therefore, height, sitting height, and sitting height/height were all considered as potential confounders. Height changed the association of BMI and WC with FVC and FEV_1_ most after inclusion in the statistical model, compared with sitting height and sitting height/height ratio. Therefore height was included in all models as confounder. Maternal educational level and pubertal development changed the regression coefficients for the association between BMI, WC and FVC and FEV_1_ > 10% and were included in the adjusted models. We also considered gas cooking, tobacco smoke exposure, asthma medication use within 48 hours before the lung function measurement, having a cold during the lung function measurement, indoor temperature and humidity as potential confounders but these did not change the association between BMI or WC and the lung function testing variables and were not included in the final models.

Differences in prevalence of baseline characteristics between boys and girls were assessed by a *t*-test.

The associations of BMI and WC with FVC, FEV_1_, and FEV_1_/FVC ratio were first explored graphically using local regression (Loess), separately for girls and boys. The associations were adjusted for confounders. This nonlinear approach resulted in smooth lines following the data without adjusting to a certain shape or predefined statistical model. This nonlinear analysis shows the shape of the relationships, but does not produce easily quantifiable estimates of the effects of BMI or WC on FVC, FEV_1_ and FEV_1_/FVC ratio. Therefore, we also conducted regression analyses of the associations between a low or high BMI and a small or large WC and FVC, FEV_1_, and FEV_1_/FVC ratio, separately for girls and boys. Statistical model: Ln (lung function testing variable) = constant + ln (height) + ln (age) + BMI_<10th percentile_ + BMI_>90th percentile_ + ‘error’. The result is the percent difference in FVC, FEV_1_ or FEV_1_/FVC ratio in children in the lowest and highest 10% of BMI compared with FVC, FEV_1_ or FEV_1_/FVC ratio in children with ‘normal’ BMI. These analyses were also conducted with WC_<10th percentile_ and WC_>90th percentile_ as exposure variable in separate models. To study possible effect modification analyses stratified for sensitization, asthma and wheeze in the past year were performed. Sensitivity analyses were performed with children with maximum difference of 150 mL between the two largest FEV_1_ measured.

Subsequently, regression analyses were performed with BMI and WC at ages 8 and 12 years and FVC, FEV_1_ and FEV_1_/FVC ratio at age 12 years. For this purpose subgroups were created; children with a high BMI or large WC at both ages (‘persistently high’), children with a high BMI or large WC at age 8 and a normal BMI or WC at age 12 years (‘high-normal’) and children with a normal BMI or WC at age 8 and a high BMI or large WC at age 12 years (‘normal-high’) were compared with children with a normal BMI or WC at both ages (‘persistently normal’). Children with a low BMI or small WC at 8 or 12 years were excluded from these analyses. These analyses were equal to the analyses previously performed regarding stratification and adjustment. Analyses were performed with SAS software version 9.3 (SAS Institute, Inc., Cary, NC).

## Results

### Study population

The study population consisted of 655 girls and 633 boys. The mean BMI was 18.6 kg/m^2^ (standard deviation (sd) 2.7) in boys and 18.9 kg/m^2^ (sd 2.7) in girls and the mean WC was 66.9 cm (sd 6.9) in boys and 66.0 cm (sd 6.5) in girls. The mean FVC was 3.2 L (sd 0.5) in girls and 3.3 L (sd 0.5) in boys, the mean FEV_1_ was 2.7 L (sd 0.4) in boys and girls. Table 1 shows the characteristics of the study population. The lung function test did not meet the inclusion criteria in 144 children, these children were somewhat younger (mean 12.5 years (sd 0.4)) than the children whose lung function measurement did meet the inclusion criteria (mean 12.7 years (sd 0.3)), but did not differ with regard to other characteristics.

**Table 1:** **Characteristics of the 12-year-old study population**

	**Girls n = 655**	**Boys n = 633**
	***Mean (sd)***	***Mean (sd)***
Age (year)*	12.7 (0.4)	12.6 (0.4)
Waist circumference (cm)*	66.0 (6.5)	66.9 (6.9)
BMI (kg/m^2^)*	18.9 (2.7)	18.6 (2.7)
Height (cm)*	160.6 (2.7)	159.2 (7.9)
Sitting height (cm)*	82.8 (4.1)	81.0 (3.7)
Sitting height/height ratio*	0.52 (0.01)	0.51 (0.01)
FEV_1_ (L)	2.73 (0.4)	2.69 (0.4)
FVC (L)*	3.19 (0.5)	3.25 (0.5)
FEV1/FVC ratio*	0.86 (0.1)	0.83 (0.1)
	*n/N (%)*	*n/N (%)*
Asthma symptoms^†^	74/636 (11.6)	93/616 (15.1)
Frequent asthma symptoms^‡^	21/636 (3.3)	33/616 (5.4)
Sensitization*	175/550 (31.8)	254/562 (45.2)
Allergic mother	219/655 (33.4)	208/633 (32.9)
Overweight^§^*	80/655 (12.2)	83/633 (13.1)
Obesity^§^*	4/655 (0.6)	7/633 (1.1)
BMI category 8 and 12 years^£^		
Normal-normal	418/563 (74.3)	393/527 (74.6)
High-normal	22/563 (3.9)	20/527 (3.8)
Normal-high	15/563 (2.7)	16/527 (3.0)
High-high	34/563 (6.0)	32/527 (6.1)
Waist circumference category 8 and 12 years^£^		
Normal-normal	403/563 (71.6)	381/527 (72.3)
High-normal	27/563 (4.8)	22/527 (4.2)
Normal-high	21/563 (3.7)	24/527 (4.6)
High-high	29/563 (5.2)	26/527 (4.9)
Maternal educational level		
Low	106/655 (16.2)	119/633 (18.8)
Intermediate	284/655 (43.5)	246/633 (38.9)
High	263/655 (40.3)	268/633 (42.3)
Use of asthma medication within 48 hours before lung function measurement	20/513 (3.9)	31/517 (6.0)
Having a cold during lung function measurement	52/510 (10.2)	44/518 (8.5)
Wheezing in the past year	25/641 (3.9)	37/617 (6.0)

*P < .05 difference between boys and girls.

^†^In the PIAMA study, ‘asthma symptoms’ are defined as at least 1 attack of wheeze, and/or at least 1 episode of dyspnoea and/or a prescription of inhaled corticosteroids in the last 12 months.

^‡^In the PIAMA study, ‘frequent asthma symptoms’ are defined as having asthma symptoms and ≥4 attacks of wheeze or ≥4 attacks of dyspnoea in the last 12 months.

^§^Overweight and obesity were defined according to standard international definitions, specified for age and gender [23].

^£^Three BMI sd-score categories and three WC sd-score categories were defined: 1) below the 10th percentile, 2) above the 90th percentile and 3) between the 10th and 90th percentile, which we call ‘normal’. The percentiles were based on the distribution for boys and girls separately, and at age 8 and 12 years separately. Using the ‘normal’ and ‘high’ categories four new categories were defined for BMI and WC separately.

Table 2 shows the distribution of the children when classified by the 10th and 90th percentile of BMI and WC in the current population. 183 children (14%) were classified differently according to WC than to BMI. All children with a BMI >90th percentile were overweight according to the international Obesity Task Force (IOTF) overweight definition [23]. In the group of children with a high BMI or large WC at age 12 years, more than half also had a high BMI or large WC at the age of 8 years.

**Table 2:** **Number of children in the different categories of waist circumference and BMI measured at age 12, for girls and boys separately**

	**BMI**							
	**Girls**				**Boys**			
**Waist circumference**	**<10** ^**th**^ **percentile**	**‘Normal’**	**>90** ^**th**^ **percentile**	**Total**	**<10** ^**th**^ **percentile**	**‘Normal’**	**>90** ^**th**^ **percentile**	**Total**
<10th percentile	31	30	0	61	27	34	0	61
‘Normal’	19	500	16	535	28	471	16	515
>90th percentile	0	21	38	59	0	19	38	57
Total	50	551	54	655	55	524	54	633

Girls: 10^th^ percentile of WC < −0.89SD, 90th percentile of WC >1.44SD; 10th percentile of BMI < −1.33SD, 90th percentile of BMI >1.44SD.

Boys: 10^th^ percentile of WC < −1.11SD, 90th percentile of WC >1.44SD; 10th percentile of BMI < −1.22SD, 90th percentile of BMI >1.67SD.

### BMI and waist circumference in relation to lung function at 12 years of age

**Figure 1:**
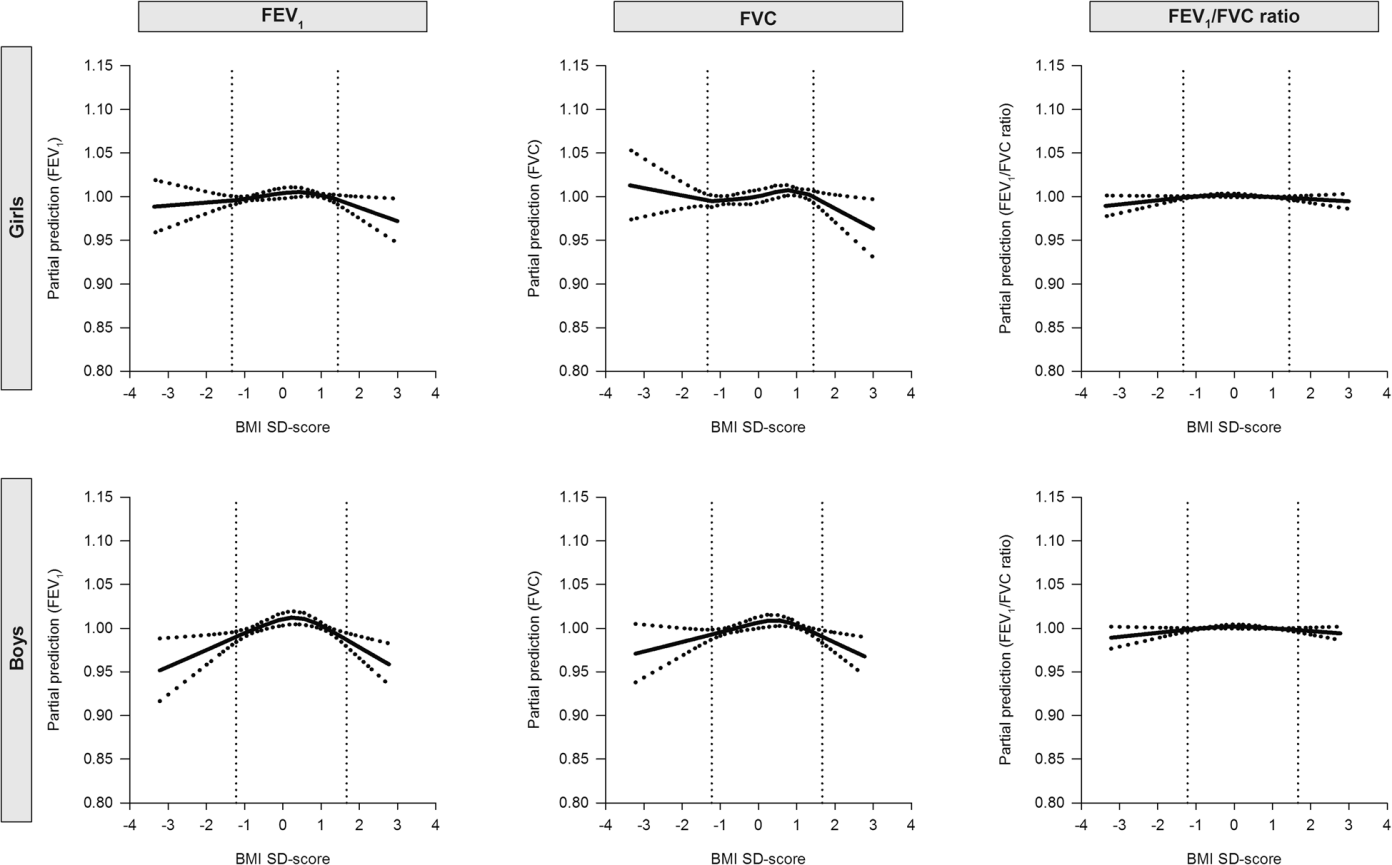
Partial predictions of FEV_1_, FVC and FEV_1_/FVC ratio by BMI SD-scores, separately for boys and girls. The lung outcomes were adjusted for age, height, pubertal development and maternal educational level. The dotted lines correspond to the 95% confidence limits.

**Figure 2:**
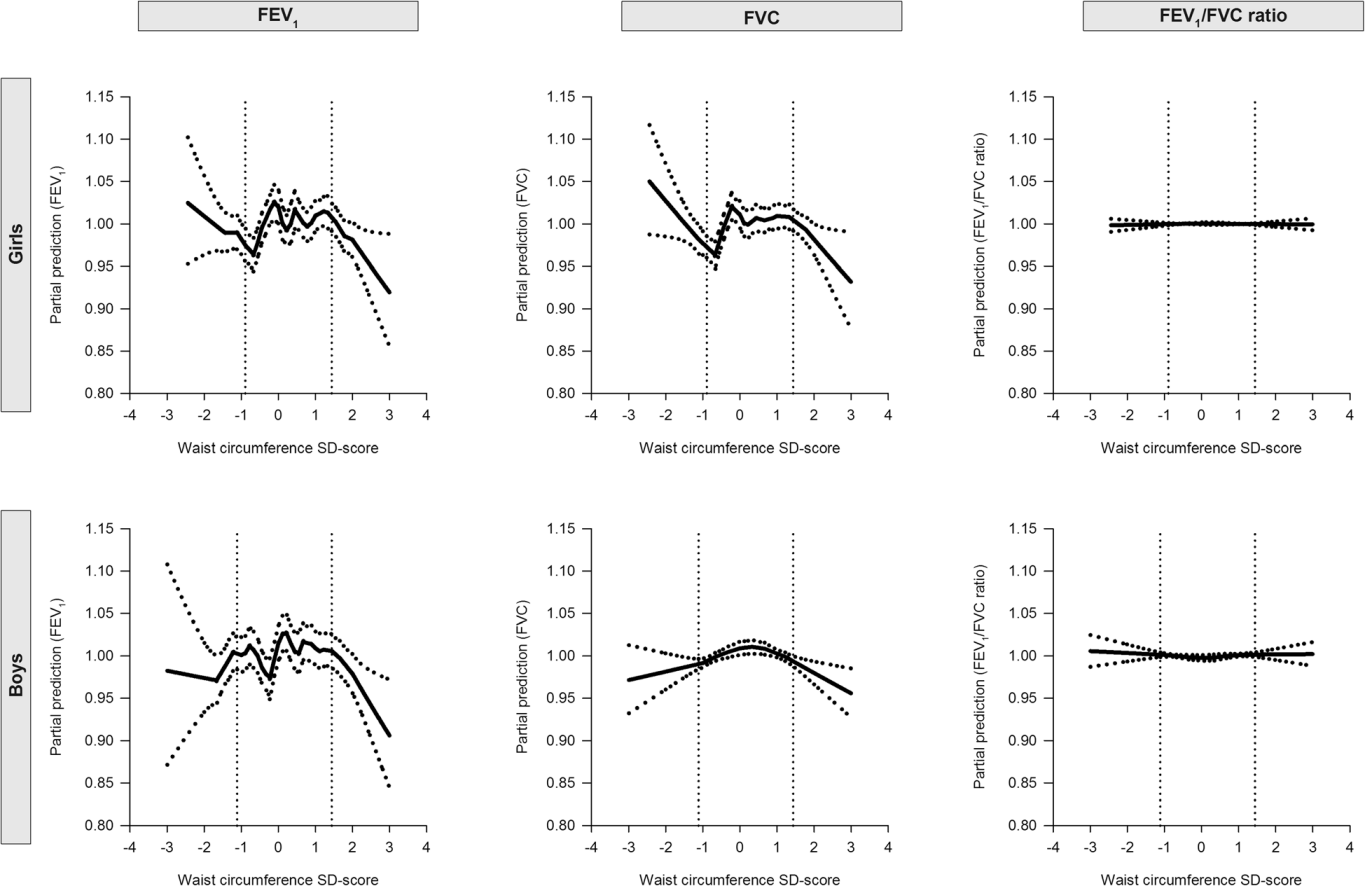
Partial predictions of FEV_1_, FVC and FEV_`1_/FVC ratio by WC SD-scores, separately for boys and girls. The lung outcomes were adjusted for age, height, pubertal development and maternal educational level. The dotted lines correspond to the 95% confidence limits.

Figures 1 and 2 illustrate the non-linear association of BMI sd-score and WC sd-score with FVC, FEV_1_ and FEV_1_/FVC ratio. No clear patterns between sd-scores of BMI and WC and FVC, FEV_1_ and FEV_1_/FVC ratio can be observed in girls. In boys, the figures suggest inverse U-shaped associations of BMI and WC sd-scores with FVC and FEV_1_. This observation would indicate that both boys with low BMI or WC sd-score and boys with high BMI or WC sd-score have lower FVC and FEV_1_ than boys with a BMI or WC sd-score in the normal range.

In the regression analyses, children with a high BMI or large WC had higher FVC than children with a normal BMI or WC. Girls with a high BMI had a 4.6% higher FVC (95% Confidence Interval (CI) 1.5, 7.9) than girls with a normal BMI in cross-sectional analyses after adjustment for confounders (Table 3). Similarly, girls with a large WC had a 3.6% higher FVC (95% CI 0.6, 6.8) than girls with a normal WC. In boys, the differences in FVC between high BMI and large WC on the one hand and normal BMI and WC on the other hand were small and not statistically significant. No statistically significant associations were observed of high BMI or large WC with FEV_1_ in girls and boys. The FEV_1_/FVC ratio was statistically significantly lower in children with a high BMI or large WC than in children with a normal BMI or WC (Table 3).

**Table 3:** **Associations of waist circumference (WC) and BMI measured at age 12 with FEV**_**1**_ **, FVC and FEV**_**1**_ **/FVC ratio separately for girls and boys**

	**FEV**_**1**_		**FVC**		**FEV**_**1**_ **/FVC ratio**	
	**Girls**	**Boys**	**Girls**	**Boys**	**Girls**	**Boys**
	**% difference**	**% difference**	**% difference**	**% difference**	**% difference**	**% difference**
	**(95% CI)**	**(95% CI)**	**(95% CI)**	**(95% CI)**	**(95% CI)**	**(95% CI)**
<10th percentile WC	**−4.3 (−7.2, −1.4)**	−2.9 (−5.9, 0.2)	**−5.6 (−8.3, −2.7)**	**−5.6 (−8.3, −2.8)**	1.1 (−0.4, 2.7)	2.3 (0.7, 4.0)
>90th percentile WC	1.7 (−1.2, 4.7)	−1.1 (−4.2, 2.1)	**4.1 (1.2, 7.2)**	2.2 (−0.7, 5.2)	**−2.0 (−3.5, −0.5)**	**−2.6 (−4.2, −1.1)**
<10th percentile BMI	**−6.9 (−9.8, −4.0)**	**−5.2 (−8.2, −2.2)**	**−6.7 (−9.5, −3.8)**	**−7.1 (−9.8, −4.4)**	−0.2 (−1.8, 1.4)	**1.7 (0.0, 3.3)**
>90th percentile BMI	2.7 (−0.3, 5.8)	−1.3 (−4.4, 1.9)	**5.1 (2.0, 8.3)**	1.5 (−1.4, 4.5)	**−2.0 (−3.5, −0.5)**	**−2.3 (−3.9, −0.7)**
Adjusted for confounders*						
<10th percentile WC	**−3.4 (−6.2, −0.4)**	−3.0 (−6.1, 0.2)	**−4.7 (−7.5, −1.8)**	**−5.6 (−8.3, −2.7)**	1.2 (−0.4, 2.8)	**2.2 (0.6, 3.9)**
>90th percentile WC	1.0 (−2.0, 4.1)	−1.3 (−4.5, 1.9)	**3.6 (0.6, 6.8)**	1.9 (−1.0, 5.0)	**−2.2 (−3.7, −0.7)**	**−2.7 (−4.2, −1.1)**
<10th percentile BMI	**−6.1 (−8.9, −3.1)**	**−5.1 (−8.2, −2.0)**	**−5.8 (−8.7, −2.9)**	**−6.9 (−9.7, −4.1)**	−0.2 (−1.8, 1.4)	1.6 (−0.1, 3.3)
>90th percentile BMI	2.0 (−1.1, 5.2)	−1.3 (−4.4, 2.0)	**4.6 (1.5, 7.9)**	1.5 (−1.4, 4.6)	**−2.2 (−3.8, −0.6)**	**−2.3 (−3.8, −0.6)**

The results are a percent difference in the lung function testing variables of the children in the lowest and the highest 10% of sd-score of waist circumference and BMI, compared with children who have a waist circumference or BMI sd-score between the 10th and 90th percentile. All analyses were adjusted for the child’s height and age.

*Adjusted for pubertal development and maternal education level.

Bold represents a statistically significant association.

Taking BMI and WC at 8 years of age into account, children with a persistently high BMI or large WC showed higher FVC at 12 years than children with a persistently normal BMI or WC. Girls with a persistently high BMI had a 5.2% higher FVC (95% CI 1.3, 9.2) than girls with persistently normal BMI (Table 4). With regard to WC, girls with persistently large WC had a 5.3% (95% CI 1.1, 9.7) higher FVC compared with girls with a persistently normal WC. In boys, the associations between persistently high BMI or large WC and FVC were not statistically significant. No statistically significant associations were observed between persistently high BMI or large WC and FEV_1_. Girls and boys with a persistently high BMI or large WC status had statistically significantly lower FEV_1_/FVC ratios (Table 4).

**Table 4:** **Prospective associations of waist circumference (WC) and BMI measured at ages 8 and 12 years with FEV**_**1**_ **, FVC and FEV**_**1**_ **/FVC ratio measured at age 12 years, separately for girls and boys**

	**FEV**_**1**_		**FVC**		**FEV**_**1**_ **/FVC ratio**	
	**Girls**	**Boys**	**Girls**	**Boys**	**Girls**	**Boys**
	**% difference**	**% difference**	**% difference**	**% difference**	**% difference**	**% difference**
	**(95% CI)**	**(95% CI)**	**(95% CI)**	**(95% CI)**	**(95% CI)**	**(95% CI)**
Waist circumference						
High-normal	4.5 (0.2, 9.0)	2.9 (−2.0, 8.0)	**4.8 (0.5, 9.3)**	3.8 (−0.7, 8.6)	−0.3 (−2.5, 1.9)	−0.8 (−3.2, 1.8)
Normal-high	1.3 (−3.4, 6.3)	−0.8 (−5.3, 3.9)	2.9 (−1.8, 8.0)	2.6 (−1.8, 7.1)	−1.4 (−3.8, 1.1)	**−2.6 (−4.9, −0.2)**
High-high	2.4 (−1.7, 6.7)	−1.3 (−5.6, 3.3)	**5.3 (1.1, 9.7)**	2.3 (−1.9, 6.6)	**−4.4 (−4.4, −0.3)**	−2.9 (−5.2, 0.6)
BMI						
High-normal	4.4 (−0.2, 9.3)	−2.7 (−7.6, 2.4)	4.0 (−0.6, 8.9)	−0.7 (−5.4, 4.2)	0.3 (−2.1, 2.7)	−1.7 (−4.3, 1.0)
Normal-high	3.9 (−1.7, 9.8)	−2.8 (−8.1, 2.8)	5.3 (−0.4, 11.2)	1.6 (−3.6, 7.1)	−1.1 (−4.0, 1.8)	**−3.4 (−6.2, −0.5)**
High-high	2.0 (−1.8, 5.9)	−0.6 (−4.6, 3.5)	**5.2 (1.3, 9.2)**	1.8 (−2.0, 5.8)	**−2.6 (−4.5, −0.7)**	**−2.1 (−4.2,-0.0)**
Adjusted for confounders*						
Waist circumference						
High-normal	**5.6 (1.2, 10.1)**	3.6 (−1.6, 9.1)	**5.7 (1.4, 10.3)**	**4.5 (0.4, 9.6)**	−0.2 (−2.3, 2.1)	−0.7 (−3.2, 2.0)
Normal-high	1.6 (−3.5, 6.9)	−1.0 (−5.6, 3.8)	2.8 (−2.3, 8.2)	2.5 (−1.9, 7.1)	−1.1 (−3.7, 1.6)	**−2.7 (−5.1, −0.3)**
High-high	1.6 (−2.7, 5.9)	−1.8 (−6.3, 2.8)	**5.0 (0.7, 9.6)**	1.7 (−2.6, 6.1)	**−2.8 (−4.9, −0.7)**	**−2.9 (−5.2, −0.5)**
BMI						
High-normal	**4.7 (0.1, 9.6)**	−3.0 (−8.2, 2.6)	4.3 (−0.4, 9.2)	−1.2 (−6.3, 4.1)	0.3 (−2.1, 2.7)	−1.4 (−4.3, 1.5)
Normal-high	**4.0 (1.6, 9.9)**	−2.5 (−7.9, 3.3)	5.6 (−0.1, 11.6)	1.8 (−3.4, 7.5)	−1.3 (−4.2, 1.6)	**−3.3 (−6.2, −0.4)**
High-high	1.3 (−2.5, 5.4)	−0.9 (−4.8, 3.3)	**4.9 (0.9, 9.1)**	1.7 (−2.2, 5.6)	**−2.9 (−4.9, −0.9)**	**−2.1 (−4.2, −0.0)**

The results are a percent difference in the lung function testing variables of the children with a high-normal, normal-high, and high-high BMI or WC status, compared with children with a normal-normal BMI or WC status. All analyses were adjusted for the child’s height and age.

*Adjusted for pubertal development and maternal education.

Bold represents a statistically significant association.

Additionally to high BMI and large WC also low BMI and small WC were studied in relation to FVC and FEV_1_. Girls and boys with a low BMI or small WC showed statistically significantly lower FVC and FEV_1_ than children with a normal BMI or WC, except for boys with a small WC in relation to FEV_1_ (Table 3).

Stratified analyses by sensitization, wheeze and asthma yielded similar results, and interaction terms between sensitization, wheeze and asthma on the one hand and BMI and WC on the other hand were mostly non-significant (see Additional file 1: Table S1). The sensitivity analyses showed that children with FEV_1_ difference <150 mL (n = 987) differed from the total group of children with FVC and FEV_1_ measurements with regard to gender, maternal education and number atopic mothers, but estimates of BMI and WC on FVC and FEV_1_ did not differ between the two groups (data not shown).

## Discussion

In a population of 12-year-old children, we examined the association of BMI and WC with lung function. Children with high BMI or large WC had a larger lung volume (FVC) than children with normal BMI or WC (statistically significant only in girls). Although the associations with FVC were statistically significant in girls and not in boys, all associations assessed were in the same direction in boys and girls. Also the children who had a high BMI or large WC already at the age of 8 years and still at the age of 12 years had a larger lung volume at age 12 than the children with normal BMI or WC at both ages. So, in children, even long term overweight seems to be associated with larger lung volume rather than smaller lung volume, which is commonly observed in overweight adults. For FEV_1_ very small (positive and negative) differences were observed between children with high BMI or large WC and children with normal BMI or WC, that were not statistically significant. The positive associations between high BMI and large WC and FVC in combination with the absence of associations with FEV_1_ resulted in lower FEV_1_/FVC ratio’s in children with high BMI or large WC (statistically significant in boys and girls). Also children who had a high BMI or large WC already at age 8 years had statistically significantly lower FEV_1_/FVC ratio’s. These observed lower FEV_1_/FVC ratios in children with high BMI or large WC may indicate obstruction of the airways.

Compared with previous studies investigating the associations of BMI and WC with FVC, FEV_1_ and FEV_1_/FVC ratio, the present study has several strengths. Our study population was relatively large, enabling stratification for gender. Furthermore, we included the child’s BMI and WC at age 8 years to examine whether persistently high BMI or large WC is important in relation FVC, FEV_1_ and FEV_1_/FVC ratio. Weight, height and WC were measured by professionals. The PIAMA birth cohort is a large cohort with almost 4000 participants at baseline. Response rates to the questionnaires are high in this study, 90% on the annual questionnaires administered between birth and age 8 years and 75% at age 11 years), but a drawback of our study is that only half of the invited subjects participated in the medical examination at 12 years. However, the children participating in the medical examination differed from the original study population only with regard to maternal education. The participating children more frequently had high-educated mothers and less frequently low-educated mothers compared with the original study population. However, we assume that the association between adiposity and lung function does not differ between children of low and high-educated parents. No differences were observed with regard to age, gender distribution, maternal allergy, and asthma symptoms between the children participating in the medical examination and the original study population. The prevalence of obesity in our study population was 1% according to the IOTF cut offs, which is similar to the obesity prevalence in the Dutch 12-year-old population [24]. This rather low obesity prevalence, compared to for instance the USA and UK, may have limited our possibilities to gain insight in the lung function of obese children. Additionally, this may explain our different findings compared with the study of Spathopoulos ea. [14] observing an inverse relation between BMI and lung function.

Our observation of lower FEV_1_/FVC ratio’s in children with high BMI or large WC is in line with previous studies on waist circumference [25] and BMI [10,12,14].

Previous studies on BMI and FVC and FEV_1_ in children examined children with a broad age range, for example 6 to 18 years, without considering specific age groups and without adjusting for pubertal development. Only Perez-Padilla et al. [12] divided their study population in children ≤ 11 years (6 to 11 years) and children > 12 years (12 to 20 years). They showed, like others, a positive association between BMI and lung function in children ≤ 11 years. However, in the children older than 12 years, their results suggest an inverse U-shaped association between BMI and FEV_1_, not with FVC, suggesting that above a BMI sd-score of 1 the FEV_1_ decreases with increasing BMI sd-score. We observed lower FEV_1_ associated with high BMI only in boys and the effect sizes were very small and not significant. The inverse U-shaped association found by Perez-Padilla et al. might be explained by the older children, as the 12-year-olds were the youngest in their age group. Our finding of a higher FVC in children with a high BMI or large WC corresponds with our previous findings in 8-year-old children and with findings of others in children [10,12,15,26]. Possibly, only in obese children, and not in moderately overweight children, the decrease in lung volume due to a high fat mass cancels out the increase of lung function with increasing BMI. During adulthood, mechanical effects of excess central adiposity reduce chest wall compliance and increase the workload of respiratory muscles, and abdominal adiposity impedes diaphragmatic descent and so reduces lung volume. In adults BMI only represents adiposity and not growth. This might explain the reduced lung volume with increasing BMI in adulthood and the increase of lung volume with increasing BMI over most of the BMI distribution in childhood. Recently, Wang and colleagues observed differing associations of BMI and body fat with lung function in 11-year-old children [13]. Like in previous studies, they showed an increase in FVC and FEV_1_ with higher BMI, but only in girls. Conversely, they observed increasing body fat being associated with reduced FVC and FEV_1_, but only in boys. They suggest a mechanical association between adiposity and lung function.

The associations between adiposity and FVC and FEV_1_ observed in our population of 12-year-olds resembled the associations we observed in the same population when they were 8 years old. These findings suggest that the transition from the ‘childhood association’ (high BMI associated with larger lung volume) to the ‘adult association’ (high BMI associated with smaller lung volume) takes place at a later age. A new wave of lung function measurements is currently taking place in the PIAMA study participants who are now 16 years old, so that we will be able to assess the association between adiposity and lung function again in 16-year-olds.

## Conclusion

At 12 years of age, a persistently high BMI or large WC is not yet associated with lower FVC and FEV_1_, suggesting that the transition from the ‘childhood association’ (high BMI associated with larger lung volume) to the ‘adult association’ (high BMI associated with smaller lung volume) takes place at a later age.

## Additional file

Additional file 1: Table S1. Associations of waist circumference (WC) and BMI with FEV_1_, FVC and FEV_1_/FVC ratio separately for girls and boys. The results are a percent difference in the lung function testing variables of the children in the lowest and the highest 10% of z-score of waist circumference and BMI, compared with children who have a waist circumference or BMI z-score between the 10^th^ and 90^th^ percentile. All analyses were adjusted for the child’s height and age.
