# Supplementary material for: BMI, waist circumference at 8 and 12 years of age and FVC and FEV1 at 12 years of age; the PIAMA birth cohort study
Source: BMC Pulm Med. 2015 Apr 22;15:39. doi: 10.1186/s12890-015-0032-0 (PMC4409985; doi:10.1186/s12890-015-0032-0)
Supplement: Additional file 1: Table S1. — Associations of waist circumference (WC) and BMI with FEV1, FVC and FEV1/FVC ratio separately for girls and boys. The results are a percent difference in the lung function testing variables of the children in the lowest and the highest 10% of z-score of waist circumference and BMI, compared with children who have a waist circumference or BMI z-score between the 10th and 90th percentile. All analyses were adjusted for the child’s height and age. [file 12890_2015_32_MOESM1_ESM.doc]

Supplement 1. Associations of waist circumference (WC) and BMI with FEV_1_, FVC and FEV_1_/FVC ratio separately for girls and boys. The results are a percent difference in the lung function testing variables of the children in the lowest and the highest 10% of z-score of waist circumference and BMI, compared with children who have a waist circumference or BMI z-score between the 10^th^ and 90^th^ percentile. All analyses were adjusted for the child’s height and age.

|  | **FEV_1_** | | | | | **FVC** | | | | **FEV_1_/FVC ratio** | | | |
| --- | --- | --- | --- | --- | --- | --- | --- | --- | --- | --- | --- | --- | --- |
|  | **Girls** | | | **Boys** | | **Girls** | | **Boys** | | **Girls** | | **Boys** | |
|  | **% difference**  **(95% CI)** | | | **% difference**  **(95% CI)** | | **% difference**  **(95% CI)** | | **% difference**  **(95% CI)** | | **% difference**  **(95% CI)** | | **% difference**  **(95% CI)** | |
| *Atopy* |  |  | |  |  |  |  |  |  |  |  |  |  |
| <10^th^ percentile WC | -4.1 | (-11.4, 3.8) | | -2.1 | -6.8, 2.9 | -6.7 | (-13.4, 0.7) | **-5.0** | **-9.5,-0.2** | 2.4 | -1.7, 6.6 | 2.5 | -0.0, 5.1 |
| >90^th^ percentile WC | -1.7 | (-7.6, 4.7) | | -1.6 | -7.1, 4.2 | -0.9 | -6.6,5.1 | 2.8 | -2.8, 8.8 | -0.8 | -3.9, 2.4 | **-3.4** | **-6.2, -0.6** |
|  |  |  | |  |  |  |  |  |  |  |  |  |  |
| <10^th^ percentile BMI | -7.2 | -13.5, -0.3 | | -5.1 | -9.9, 0.0 | **-6.9** | **-13.1, -0.3** | **-7.1** | **-11.8, -2.3** | -0.2 | -3.8,3.6 | 1.8 | -0.9, 4.6 |
| >90^th^ percentile BMI | -5.0 | -10.9, 1.2 | | -1.2 | -6.5,4.4 | -2.2 | -8.0, 3.9 | 1.6 | -3.8, 7.2 | -2.5 | -5.6, 0.8 | -2.2 | -5.0, 0.6 |
| *No atopy* | | |  | | | | | | | | | | |
| <10^th^ percentile WC | -2.2 | (-5.9, 1.1) | | -0.0 | -7.3, 2.2 | **-3.8** | **-7.2, -0.2** | **-5.2** | **-9.2, -1.0** | 1.2 | -0.9, 3.2 | 2.2 | -0.3, 4.7 |
| >90^th^ percentile WC | 1.7 | (-1.8, 5.3) | | 0.0 | -4.1, 4.8 | **5.1** | **1.4, 8.8** | 2.0 | -2.0, 6.1 | **-2.7** | **-4.6,-0.8** | -1.4 | -3.6, 0.8 |
|  | | | | | | | | | | | | | |
| <10^th^ percentile BMI | **-4.7** | **-8.2, -1.0** | | **-5.0** | **-9.6, -0.0** | **-4.3** | **-7.9, -0.6** | **-6.3** | **-10.4,-2.0** | -0.3 | -2.5, 1.9 | 1.2 | -1.3, 3.7 |
| >90^th^ percentile BMI | **4.3** | **0.6, 8.1** | | -1.6 | -6.1, 3.1 | **6.9** | **3.0, 10.8** | 1.3 | -2.8, 5.6 | **-2.1** | **-4.1,-0.1** | **-2.4** | **-4.6, -0.1** |
|  |  |  | |  |  |  |  |  |  |  |  |  |  |
| *Asthma* |  |  | |  |  |  |  |  |  |  |  |  |  |
| <10^th^ percentile WC | -1.8 | -11.7, 9.3 | | 5.1 | -5.8, 17.2 | -3.9 | -11.2, 3.9 | 0.2 | -9.1, 10.5 | 2.0 | -3.6, 8.0 | 3.5 | -2.1, 9.4 |
| >90^th^ percentile WC | 0.3 | -9.5, 11.3 | | -6.0 | -15.4, 4.4 | 4.3 | -3.3, 12.5 | -3.1 | -11.8, 6.5 | -3.3 | -8.4, 2.2 | -2.6 | -7.7, 2.8 |
|  |  |  | |  |  |  |  |  |  |  |  |  |  |
| <10^th^ percentile BMI | -7.3 | -15.6, 1.8 | | -0.3 | -11.6, 12.6 | **-7.2** | **-13.2, -0.8** | -3.3 | -13.2, 7.7 | 0.0 | -5.0, 5.3 | 2.3 | -3.8, 8.9 |
| >90^th^ percentile BMI | 5.6 | -4.9, 17.4 | | -6.4 | -16.7, 5.2 | 7.6 | -0.2,16.1 | -5.1 | -14.5, 5.2 | -1.8 | -7.3,4.1 | -1.3 | -7.0, 4.8 |
|  |  |  | |  |  |  |  |  |  |  |  |  |  |
| *No asthma* |  |  | |  |  |  |  |  |  |  |  |  |  |
| <10^th^ percentile WC | **-3.7** | **-6.6, -0.6** | | **-4.1** | **-7.2, -0.9** | **-4.7** | **-7.8,-1.6** | **-6.4** | **-9.2, -3.5** | 1.0 | -0.7,2.6 | **2.1** | **0.4, 3.8** |
| >90^th^ percentile WC | 1.4 | -1.7,4.7 | | -0.9 | -4.0, 2.4 | **3.8** | **0.5, 7.3** | 2.6 | -0.5, 5.8 | **-2.0** | **-3.6, -0.4** | **-2.8** | **-4.3, -1.1** |
|  |  |  | |  |  |  |  |  |  |  |  |  |  |
| <10^th^ percentile BMI | **-5.7** | **-8.7,-2.5** | | **-5.9** | **-8.9, -2.8** | **-5.3** | **-8.6, -2.0** | **-7.4** | **-10.2, -4.5** | -0.3 | -2.0, 1.4 | 1.4 | -0.3, 3.1 |
| >90^th^ percentile BMI | 1.9 | -1.3,5.3 | | -1.0 | -4.1 ,2.3 | **4.7** | **1.2, 8.2** | 2.4 | -0.7, 5.5 | **-2.3** | **-3.9,-0.6** | **-2.6** | **-4.2,-1.0** |
|  |  |  | |  |  |  |  |  |  |  |  |  |  |
| *Wheeze* |  |  | |  |  |  |  |  |  |  |  |  |  |
| <10^th^ percentile WC | -3.7 | -16.3, 10.9 | | -1.2 | -17.6, 18.4 | **-13.3** | **-23.3, -2.1** | 0.4 | -15.3, 19.2 | 9.2 | -0.7, 20.0 | -2.0 | -10.9, 7.9 |
| >90^th^ percentile WC | -6.1 | -22.3, 13.5 | | **-20.7** | **-36.2, -1.5** | 3.8* | -12.0, 22.3 | -17.6 | -32.9, 1.1 | -8.0 | -19.1, 4.5 | -3.1 | -13.6, 8.7 |
|  |  |  | |  |  |  |  |  |  |  |  |  |  |
| <10^th^ percentile BMI | -0.4 | -12.6, 13.6 | | -20.6 | -41.2, 7.4 | **-11.4** | **-21.0, -0.5** | -18.7 | -38.9, 8.0 | **10.2** | **0.6, 20.7** | -2.3 | -17.3, 15.3 |
| >90^th^ percentile BMI | -9.2 | -28.0, 14.5 | | **-21.2** | **-36.1, -3.0** | -13.6 | -29.5, 5.9 | **-18.1** | **-32.7,-0.2** | 4.1 | -11.3, 22.3 | -3.2 | -13.7, 8.6 |
|  |  |  | |  |  |  |  |  |  |  |  |  |  |
| *No wheeze* |  |  | |  |  |  |  |  |  |  |  |  |  |
| <10^th^ percentile WC | **-3.4** | **-6.3, -0.4** | | -2.9 | -6.0, 0.3 | **-4.5** | **-7.4, -1.4** | **-5.8** | **-8.6, -2.9** | 1.0 | -0.6, 2.6 | **2.5** | **0.9,4.3** |
| >90^th^ percentile WC | 1.1 | -1.9, 4.3 | | -0.8 | -3.9, 2.4 | **3.7** | **0.6, 7.0** | 2.6 | -0.4, 5.7 | **-2.1** | **-3.7, -0.6** | **-2.7** | **-4.3, -1.1** |
|  |  |  | |  |  |  |  |  |  |  |  |  |  |
| <10^th^ percentile BMI | **-6.0** | **-9.0, -3.0** | | **-5.1** | **-8.1, -2.0** | **-5.6** | **-8.7, -2.5** | **-6.8** | **-9.6, -4.0** | -0.4 | -2.0, 1.3 | **1.5** | **-0.2, 3.2** |
| >90^th^ percentile BMI | 2.2 | -1.0, 5.4 | | -0.9 | -4.0, 2.4 | **4.9** | **1.6, 8.2** | 2.1* | -0.9, 5.2 | **-2.2** | **-3.8, -0.7** | **-2.4** | **-4.0, -0.8** |

* Interaction term is significant (*P*<.05)

**Bold** represents significant associations.

Only the interaction terms of large WC and wheeze in girls and high BMI and wheeze in boys in the association with FVC were statistically significant. Girls with a large WC had higher FVC than girls with normal WC, but this association was only statistically significant in the non-wheezers. In boys, the association between high BMI and FVC wheezers had statistically significant lower FVC, whereas non-wheezers had higher FVC (NS).
